# Typing of hereditary renal amyloidosis presenting with isolated glomerular amyloid deposition

**DOI:** 10.1186/s12882-019-1667-5

**Published:** 2019-12-23

**Authors:** Danyang Li, Dan Liu, Hui Xu, Xiao-juan Yu, Fu-de Zhou, Ming-hui Zhao, Su-xia Wang

**Affiliations:** 1 Laboratory of Electron Microscopy, Pathological Center, Peking University First Hospital, Beijing, 100034 People’s Republic of China; 2 Proteomics Laboratory, Medical and Healthy Analytical Center, Peking University Health Science Center, Beijing, 100191 People’s Republic of China; 3 Renal Division, Department of Medicine, Peking University First Hospital; Institute of Nephrology, Peking University; Key Laboratory of Renal Disease, Ministry of Health of China; Key Laboratory of CKD Prevention and Treatment, Ministry of Education of China, Beijing, 100034 People’s Republic of China

**Keywords:** Hereditary amyloidosis, Kidney, Mass spectrometry, Gene mutation, Immunohistochemistry

## Abstract

**Background:**

The commonly used methods for amyloid typing include immunofluorescence or immunohistochemistry (IHC), which sometimes may come with diagnostic pitfalls. Mass spectrometry (MS)-based proteomics has been recognized as a reliable technique in amyloid typing.

**Case presentation:**

We reported two middle-aged patients who presented with proteinuria, hypertension and normal renal function, and both had a family history of renal diseases. The renal biopsies of both patients revealed renal amyloidosis with the similar pattern by massive exclusively glomerular amyloid deposition. The IHC was performed by using a panel of antibodies against the common types of systemic amyloidosis, and demonstrated co-deposition of fibrinogen Aα chain and apolipoprotein A-I in the glomerular amyloid deposits of each patient. Then the MS on amyloid deposits captured by laser microdissection (LMD/MS) and genetic study of gene mutations were investigated. The large spectra corresponding to ApoA-I in case 1, and fibrinogen Aα chain in case 2 were identified by LMD/MS respectively. Further analysis of genomic DNA mutations demonstrated a heterozygous mutation of p. Trp74Arg in ApoA-I in case 1, and a heterozygous mutation of p. Arg547GlyfsTer21 in fibrinogen Aα chain in case 2.

**Conclusions:**

The current study revealed that IHC was not reliable for accurate amyloid typing, and that MS-based proteomics and genetic analysis were essential for typing of hereditary amyloidosis.

## Background

Amyloidosis is a protein misfolding disorder, in which normally soluble proteins undergo conformational changes and are aggregated abnormally as insoluble fibrils deposited in the extracellular space, resulting in structural and functional damage of multiple organs [1]. Renal amyloidosis is a frequent manifestation of systemic amyloidosis, and may cause end-stage renal disease (ESRD). Currently 36 precursor proteins have been associated with amyloidosis. The common types of systemic amyloidosis include immunoglobulin light chain amyloidosis (AL), amyloid A amyloidosis (AA) and leukocyte chemotactic factor 2 (Lect2) amyloidosis [2]. However, hereditary amyloidosis including transthyretin, fibrinogen Aα chain, apolipoprotein A-I and apolipoprotein A-II, lysozyme, gelsolin, and cystatin C types have been reported in the kidney [3–7].

The involved organs vary in different types of hereditary amyloidosis. Transthyretin amyloidosis affects mainly peripheral and autonomic nervous systems, with invariable cardiac involvement, and rare kidney involvement [8]; while fibrinogen Aα chain, ApoA-I and ApoA-II, lysozyme amyloidosis is generally non-neuropathic with prominent renal involvement [9]. It has been reported that fibrinogen Aα chain amyloidosis (AFib) was the most common type of hereditary renal amyloidosis, and usually presents with heavy proteinuria or nephrotic syndrome, with exclusive glomerular amyloid deposition [10]. ApoA-I amyloidosis (AApoA-I) affects the kidneys, liver, heart, and other systems, and the main location of ApoA-I amyloid deposition in renal parenchyma is the medullary interstitium rather than the glomeruli [11].

Typing of amyloidosis is necessary for therapy and prognosis. Immunofluorescence (IF) and immunohistochemistry (IHC) are the commonly used methods for amyloid typing, but there are potential diagnostic pitfalls giving rise to false negative or misleading results [12]. Laser microdissection and mass spectrometry (LMD/MS)-based proteomic analysis has emerged as a new technique for amyloid classification [13]. Here we describe two unusual cases presenting with isolated glomerular amyloid deposits. Initial classification was inconclusive or even misleading by IHC alone, and acquired accurate typing by LMD/MS analysis and genetic testing.

## Case presentation

### Case 1

A 40-year-old Chinese Han-ethnic man presented with ankle and eyelid edema, proteinuria (urinary protein excretion was 3.92 g/24 h) and hypertension for one month, His father died of nephrotic syndrome at the age of 60 years without renal biopsy.

Laboratory tests showed no monoclonal gammopathy in his serum and urine. He had hypoalbuminemia (31.0 g/L), normal serum creatinine (69.30 μmol/L), and low plasma levels of HDL (0.50 mmol/L). He did not have either macroglossia or cutaneous bleeding, but he presented with hepatomegaly (15.7 cm) and splenomegaly (13.7 cm) by abdominal ultrasonography. Electrocardiogram revealed sinus bradycardia, left ventricular high voltage, and flat T wave, but echocardiogram was normal. (The main clinical characteristics and laboratory findings are attached in the Additional file 1).

**Fig. 1:**
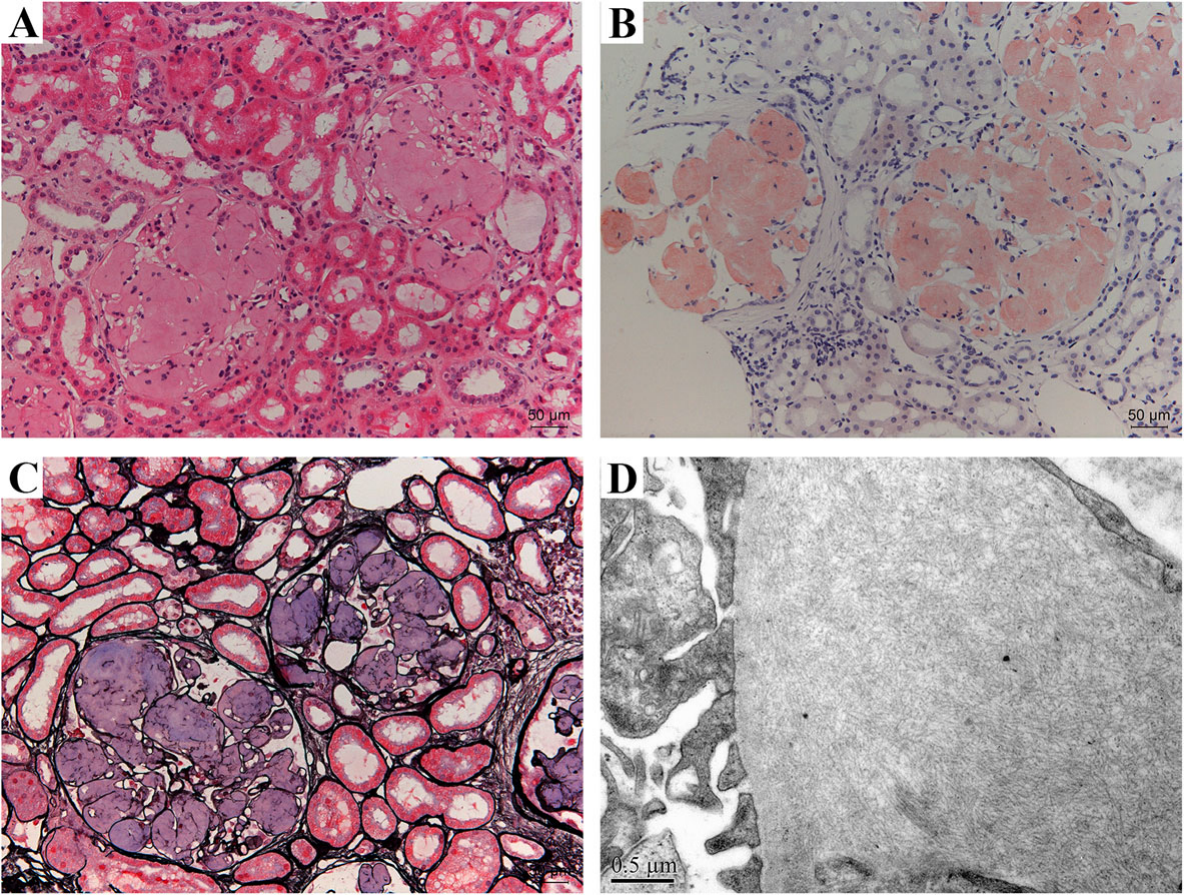
Kidney biopsy findings. The glomerular architecture was destroyed, and replaced with massive amorphous eosinophilic deposits (**a**, hematoxylin and eosin stain), which exhibited positive Congo red stain (**b**) and located in the mesangium and subendothelia of glomeruli (**c**, periodic acid-silver methenamine). EM demonstrated randomly arranged nonbranching fibrils in a diameter of 8–12 nm in glomerular subendothelial area(**d**). Magnification, × 200 in A-C; × 40,000 in D

The renal biopsy showed there were 55 glomeruli in the specimen for light microscopy (LM), and extensive amyloid deposits exclusively in the glomeruli were identified, which produced the apple-green birefringence of Congo red staining under polarized light. No amyloid deposit was identified in the tubulointerstitium and vascular walls. Routine IF examination showed negative staining for immunoglobulins, complements, and light chains (κ, λ). EM demonstrated randomly arranged fibrils with a diameter of 8–12 nm deposited in mesangium and subendothelial area (Fig. 1).

**Fig. 2:**
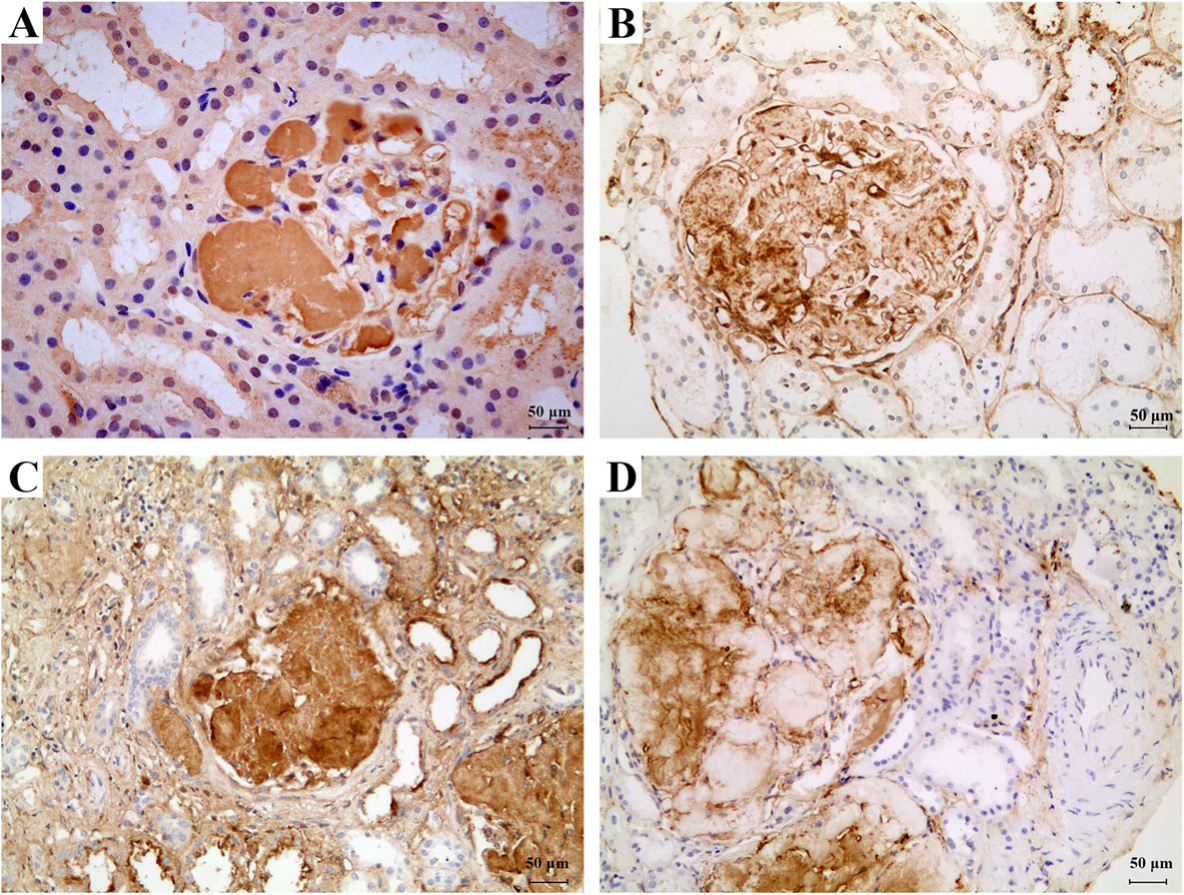
IHC results for ApoA-I and fibrinogen Aα in the two cases. The glomerular amyloid deposits were strong positive for ApoA-I in case 1 (**a**) and case 2 (**c**); but were weakly, unevenly positive for fibrinogen Aα in case 1 (**b**) and case 2 (**d**) respectively. Magnification, × 200 in A- D

The IHC typing of renal amyloid initially using an incomplete panel of antibodies directed against light chains (κ, λ), amyloid A, and fibrinogen Aα chain was performed, and a preliminary diagnosis of probably AFib was considered according to the weak positive staining of fibrinogen Aα and its characteristic feature of exclusive glomerular amyloid deposition. Then we have performed a detailed IHC study using 8 types of the common systemic amyloid precursors. It showed a strong and uniform positive staining for ApoA-I, and weak, spotty staining for fibrinogen Aα, in the glomerular amyloid deposits in case 1 (Fig. 2a-b). The IHC for lysozyme, transthyretin, Lect2, or AA were all negative.

**Fig. 3:**
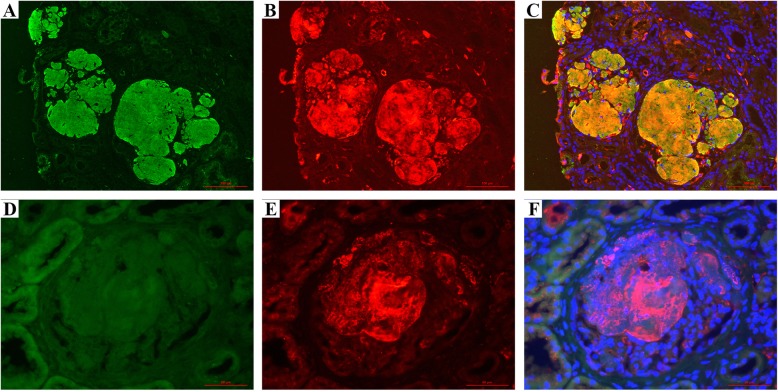
Double IF staining for ApoA-I and fibrinogen Aα in the two cases. The glomerular amyloid deposits were positive for both of ApoA-I (**a**), and fibrinogen Aα (**b**), and indicated in the merged image (**c**) in Case 1. The glomerular amyloid deposits were negative for ApoA-I (**d**), but positive for fibrinogen Aα (**e**) and indicated in the merged image (**f**) in case 2. Magnification, × 200 in A-C; × 400 in D-F

Double IF labeling for ApoA-I and fibrinogen Aα chain showed a strong and even staining for ApoA-I, but uneven staining for fibrinogen Aα chain in the glomerular amyloid deposits. The coexistence of ApoA-I and fibrinogen Aα chain in the majority of amyloid deposits was observed in the merged image (Fig. 3a-c), which suggested a mixed type of both ApoA-I and fibrinogen Aα chain in this patient.

**Fig. 4:**
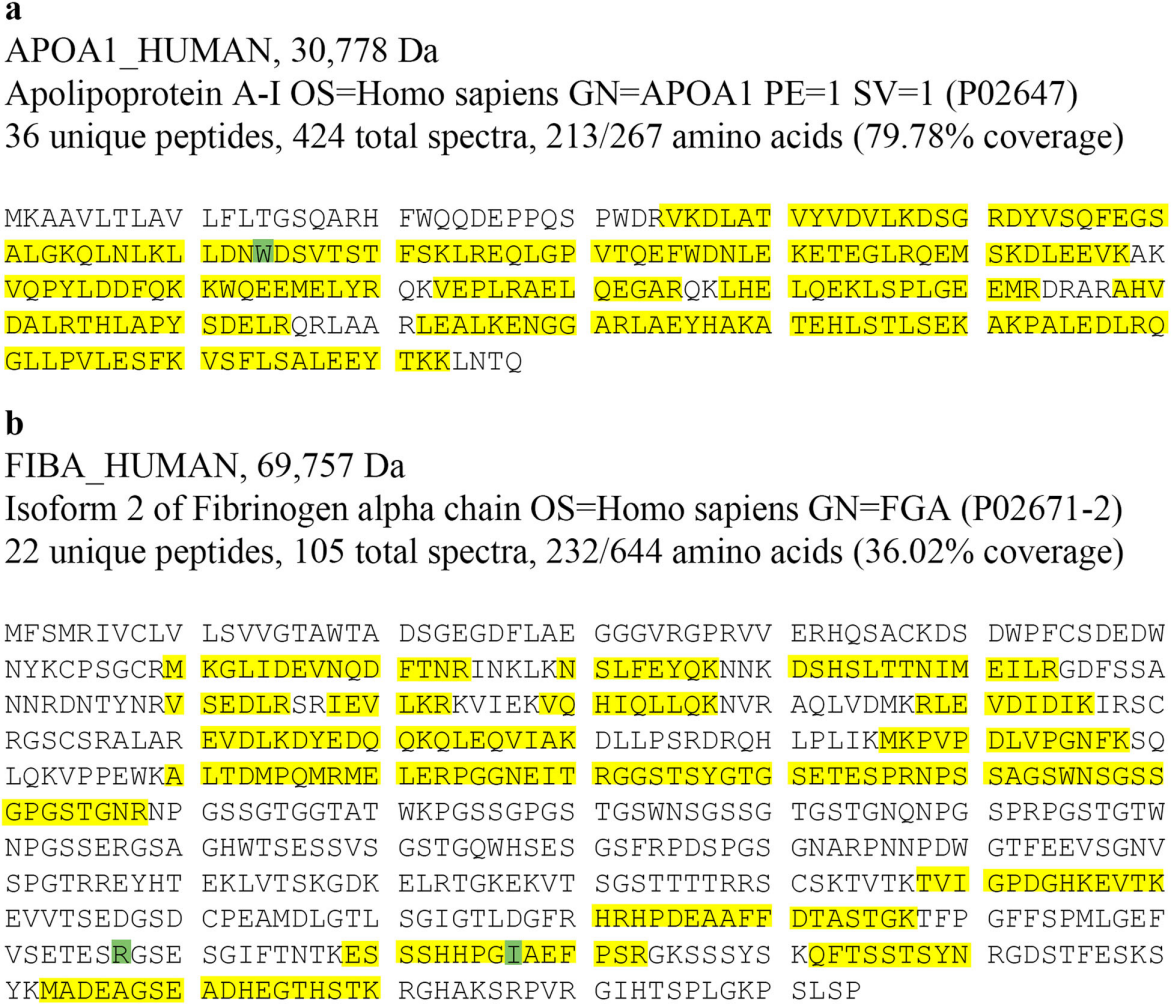
The percentage of representative sequence (peptide) coverage of protein detected by LC-MS/MS. (**a**) 36 unique peptides for 79.78% coverage of ApoA-I with > 99% probability in case 1; (**b**) 22 unique peptides for 36.02% coverage of fibrinogen Aα with > 99% probability in case 2. The green letter (W) in (a) is at residue 74 of the wild-type ApoA-I, and that (R, I) in (b) are at residue 547 and 567 of the wild type fibrinogen Aα

We have performed the LMD/MS analysis according to the previous method [13]. It showed large spectra of Apolipoprotein E protein and serum Amyloid P component ranging from 27 to 118, which are common constituents of amyloid. The most abundant peptides detected was ApoA-I with 424 spectra, 36 unique peptides for 79.78% coverage of ApoA-I (Fig. 4a), but the content of fibrinogen Aα chain was quite a few with only 20 spectra, 15 unique peptides for 26.55% coverage of fibrinogen Aα, which is also present in the normal glomeruli (day 0 protocol transplant kidney biopsies). (MS data by spectra was attached in Additional file 2).

**Fig. 5:**
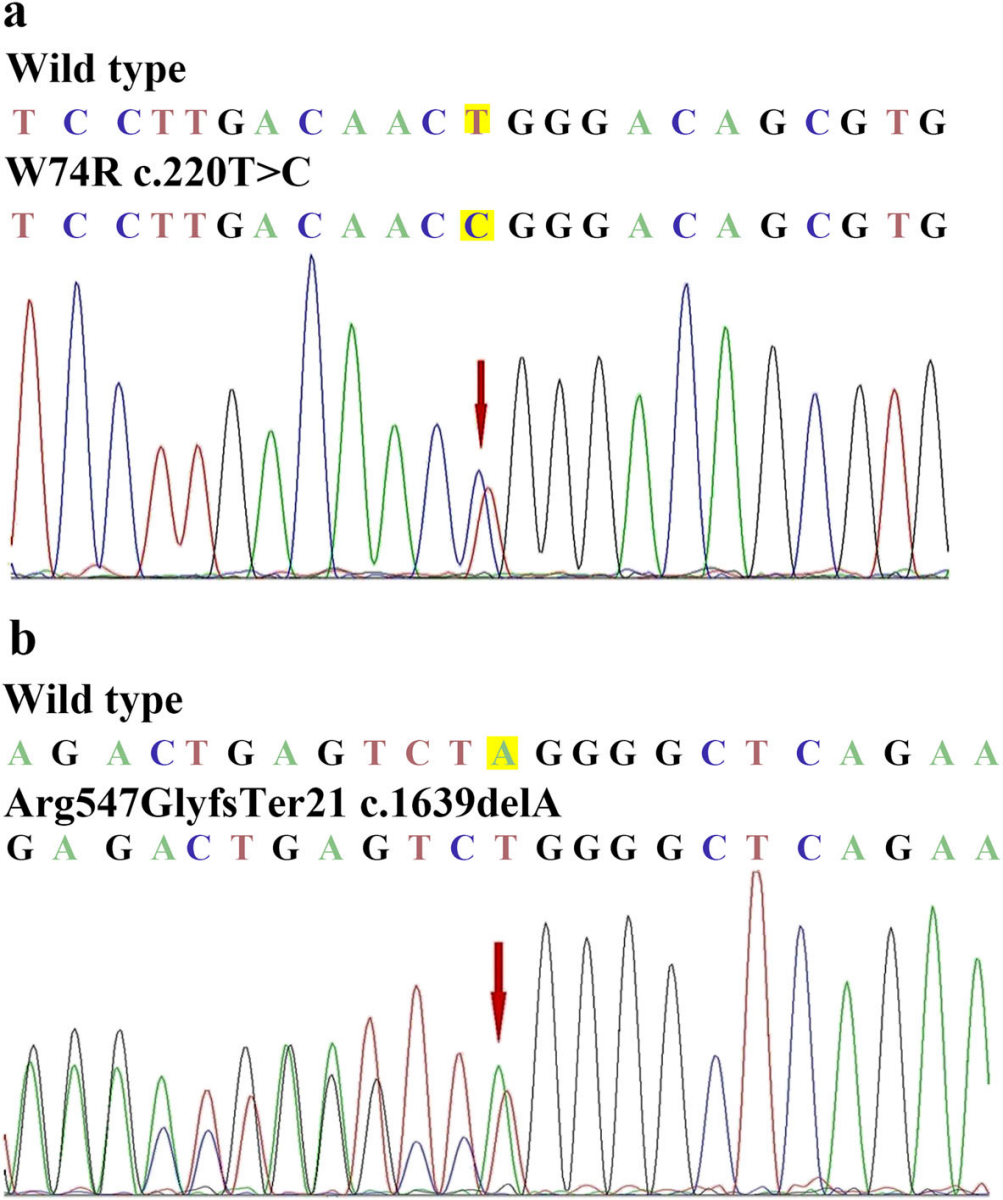
(**a**) The mutation in the APOAI gene of case1, c.220 T > C (red arrow indicates the location of mutant), which resulted in amyloidogenic Trp74Arg variant of ApoA-I. (**b**) The mutation in the fibrinogen gene of case2, c.1639delA (red arrow indicates the location of mutant), which resulted in an amyloidogenic p. Arg547GlyfsTer21 variant of fibrinogen

Genetic analysis showed a heterozygous mutation in c.220 T > C substitution in the APOA1 gene, leading to the replacement of tryptophan by arginine at residue 74 (p. Trp74Arg) (Fig. 5a). No gene mutation of fibrinogen Aα chain was detected in this patient. The final diagnosis of AApoA-I in case 1was confirmed.

### Case 2

A 47-year-old Chinese Han-ethnic man who had suffered intermittent eyelid edema for 10 months without known kidney disease, presented with hypertension, nephrotic range proteinuria (urinary protein, 3.6 g/24 h; serum albumin, 33.2 g/L; total cholesterol, 9.78 mmol/L; low density lipoprotein, 6.93 mmol/L) and normal renal function with a serum creatinine of 93.4 μmol/L. The patient’s mother had an unknown renal disease and died of uremia.

On physical examination, BP was 196/120 mmHg, there were no signs of neuropathy, autonomic dysfunction, cardiomyopathy or gastroenteropathy. Laboratory tests showed no anemia or thrombocytopenia, liver function tests and blood coagulation tests were normal with plasma fibrinogen concentration of 2.237 g/L (reference: 1.5–4 g/L). No monoclonal immunoglobulin in serum or urine was detected. Lower extremity vascular ultrasound showed the mild thickened intima with small plaque in the lower extremity artery. (The main clinical characteristics and laboratory findings are attached in the Additional file 1).

The renal biopsy contained 16 glomeruli in specimen for LM, which also showed the similar histological features by extensive amyloid deposits exclusively in the glomeruli, no amyloid deposits in the tubulointerstitium and vascular walls. Routine IF examination showed negative staining for immunoglobulins, complements, and light chains (κ, λ).

The IHC exhibited a strong and uniform positive staining for ApoA-I, and weak, spotty staining for fibrinogen Aα, in the glomerular amyloid deposits in case 2 (Fig. 2c-d), but negative for lysozyme, transthyretin, Lect2, or AA.

Double IF staining showed a strong, uneven staining for fibrinogen Aα chain, but negative staining for ApoA-I in the glomerular amyloid deposits (Fig. 3d-f), which is different from the result by IHC, and suggested the probability of fibrinogen Aα chain amyloid in case 2.

LMD/MS showed a high content of fibrinogen Aα chain with 105 spectra, 22 unique peptides for 36.02% coverage of fibrinogen Aα chain (Fig. 4b), no spectra for intact ApoA-I was detected, but quite a few truncated ApoA-I existing with only 4 spectra, 3 unique peptides for 13.88% coverage. (MS data by spectra was attached in Additional file 2).

Genetic analysis of the case 2 showed a heterozygous mutation of a single nucleotide deletion at position 1639 of the fibrinogen Aα chain gene that gives a frame-shift at codon 547(c.1639delA) and premature termination at codon 567 (p. Arg547GlyfsTer21), but without gene mutation of ApoA-I (Fig. 5b). The typing of AFib was diagnosed in case 2 finally.

## Discussion and conclusions

We here described two middle-aged Chinese patients with renal hereditary AApoA-I and AFib, respectively. Both of them presented with heavy proteinuria, hypertension and normal renal function, and a family history of kidney disease. Histology of renal biopsies of both cases showed the similar feature of exclusive glomerular amyloid deposits without tubulointerstitial and vascular amyloid deposition, which suggested the type of AFib as usually. The IHC on paraffin sections showed positive staining for both of fibrinogen Aα chain and ApoA-I in these two patients; further double-IF staining for fibrinogen Aα chain and ApoA-I identified the coexistence of two types of AFib and AApoA-I in case 1, while only positive staining for fibrinogen Aα chain in case 2. The MS-based proteomic analysis suggested the major component of amyloid as ApoA-I in case 1, and fibrinogen Aα chain in case 2, which were in accordance with the genetic testing of case 1 with a heterozygous mutation of p. Trp74Arg in ApoA-I, and case 2 with a heterozygous mutation of p. Arg547GlyfsTer21 in fibrinogen Aα chain, respectively. Hence the isolated glomerular amyloid deposition can be caused by hereditary AFib as well as AApoA-I.

The accurate typing of amyloidosis is challenging for the rare types of amyloidosis. In our study of case 1, the incomplete panel of antibodies to amyloid precursors missed the specific type of AApoA-I, and led to the misdiagnosis initially. Although we have performed a detailed study using a complete panel of antibodies to 8 types of systemic amyloid precursors by IHC, the positive staining for both of ApoA-I and fibrinogen Aα chain on glomerular amyloid deposition were detected in these two patients. How to distinguish the specific labeling of amyloid proteins from nonspecific background is challenging. It has been emphasized that IHC or IF may cause diagnostic pitfalls in amyloid typing [12, 14]. There are several reasons assumed to cause the false negative or nonspecific staining in amyloid typing by IHC or IF alone. Firstly, antigen masking due to the abnormal folding of polypeptides of amyloid precursors and its combination with other components of amyloid P, Apolipoprotein E and glycosaminoglycan in the amyloid deposits may block the recognition of antigen epitope by antibodies. Secondly, the main constitution of amyloid fibrils is some fragments of proteins other than the whole molecules, such as the variable region of immunoglobulin light chains in AL, and N-terminal residues of 1–96 amino acids in ApoA-I, while the commercial reagents provide antibodies against the constant region of immunoglobulin light chains or the epitopes of antigens which are not included in the amyloid deposits. Thirdly, the nonspecific staining of amyloid deposits may be caused by the following conditions: trapping of serum proteins leading to positive staining of light chains, stickiness of amyloid deposits with increased affinity to other proteins [15], especially in immunoperoxidase method on paraffin-embedded sections with highly background, such as the weak positive staining of fibrinogen Aα in Case 1 should have been considered non-diagnostic and possibly spurious (amyloid is “sticky”). Finally, reasonable control assays and the interpretation of results are essentially important. The positive control tissues to validate the specificity of antibodies and the efficient workflow for immunolabeling methods should be set up, especially for rare types of amyloid. The specific diagnostic labeling shows a strong, evenly uniform staining, which distributed in the area of amyloid deposits, while the nonspecific background displayed as weak, unevenly spotty, diffuse staining [14]. Sometimes the clinical relevance of certain amyloids may provide a reference clue for amyloid typing. Amyloidosis derived from immunoglobulin light chain, or heavy chain usually have serum or/and urine monoclonal gammopathy [16], hereditary amyloidosis may have a familial history of amyloidosis, but there are exceptions for this principle. Lachman et al. have reported that about 10% of renal amyloid patients with monoclonal gammopathy presumptively were diagnosed as AL amyloidosis initially, which belonged to hereditary amyloidosis at last [17].

Recently MS-based proteomics have proved to be valuable to determine the nature and type of the amyloid protein in the challenging cases that could not be typed by routine IHC [13]. In the current study, we performed LMD/MS-based proteomic analysis and gene mutation testing to make the correct amyloid typing in these two patients finally. However, LMD/MS analysis in the previous reports and our study were based on qualitative data rather than quantitative result [13, 18]. Generally, a higher number of mass spectra and a higher protein probability score of identified proteins indicated not only greater abundance but also a higher confidence in the protein identification. The protein ranked in a high probability score on the list of identified proteins was determined as the precursors of amyloid. Otherwise, it was recognized as negative result when it had a few spectra similar to the background of normal control tissue, and may be caused by blood contamination. In addition, the detection of gene mutations corresponding to the precursor proteins provided further validation for familial hereditary amyloidosis.

AApoA-I is a rare type of hereditary systemic amyloidosis associated with mutations in the APOA1 gene, and in an autosomal dominant inheritance with a complete penetrance [4]. ApoA-I is a 28 kDa plasma protein synthesized by liver and intestine, which constitutes the high-density lipoprotein particle (HDL) and participates the esterification of cholesterol. More than half of AApoA-I patients show the decreased plasma HDL and hyperlipidemia [11, 19]. The N-terminal fragments of ApoA-I are the main components of amyloid deposits [4]. The affected organs include liver, kidney, larynx, skin, heart, spleen, peripheral nerve, etc. It has been reported that the diversity of involved organs may relate to the site of the APOA1 mutation, patients with alterations in codons of 1 to 75 mainly suffer from hepatic, splenic and renal amyloidosis, while mutations affecting residues 173 to 178 mainly cause amyloidosis of the heart, peripheral nerve, larynx, and skin [11, 20]. Renal involvement of AApoA-I is quite common, usually presented with tubulointeretitial disease, and demonstrated medullary interstitial amyloid deposits [11]; rare cases show glomerular and vascular amyloid deposits [20–23]. In the current study of case 1 with a heterozygous mutation of p. Trp74Arg in ApoA-I, presented with mild to moderate proteinuria, low plasma HDL clinically, and isolated glomerular amyloid deposition histologically, which manifested as the rarely phenotype of AApoA-I.

AFib is an autosomal dominant hereditary amyloidosis, caused by the mutations in the coding region of the fibrinogen Aα chain gene [3]. The penetrance of AFib is variable, nearly half of AFib patients lack a familial history of renal diseases and amyloidosis [24]. The onset age of AFib patients usually begin from the fourth to the ninth decade of life, with a median age of 58 years [25]. The clinical manifestation of AFib is kidney dominant presented with proteinuria, progressive renal impairment, and hypertension. The striking pathological feature of AFib is massive and exclusive glomerular amyloid deposits without vascular and interstitial amyloid deposition [10]. Most of AFib patients have no evidence of amyloid cardiomyopathy, except for a recently reported patient with AFib, who underwent a combined liver and kidney transplantation, and found amyloid deposits in carotid atheromatous plaque and endomyocardial vessels [26]. The other extrarenal involved organs including liver, spleen, adrenal gland and peripheral neural system are rarely reported [24, 27]. There is a hot mutation of fibrinogen Aα chain by a heterozygous mutation of single base substitution that alter the codon at position of 526 from glutamic acid to valine(E526V) detected in about 90% of AFib patients, other variants include the single base substitutions (E540V, P552H, T538K, R554L), the frameshift mutations (4904delG and 4897delT), and an insertion/deletion mutation (1636-1650del, 1649-1650insCA) [24, 27]. The case 2 have found a de novo mutation of fibrinogen Aα chain gene with a single base deletion resulting in a frame-shift at codon 547 and premature termination at codon 567 (p. Arg547GlyfsTer21) in this Chinese AFib patient, which expanded the patterns of gene mutations in fibrinogen Aα chain.

The progression of hereditary amyloidosis is slowly when there is no cardiac amyloidosis. The median time from the onset of proteinuria to end-stage renal disease (ESRD) in AFib is 4.6 years, is substantially faster than that of AApoA-I, in which it is approximately 8 years [24, 28]. Kidney transplantation is a therapeutic option for hereditary renal amyloidosis, but may be confronted with a recurrence of amyloid in the graft kidney. This contrasts with the better long outcome by a combined liver and kidney transplantation, whereby elimination of the source of the circulating amyloidogenic variants prevents recurrence of amyloid deposition [26].

In this study, we described two rare cases of renal hereditary amyloidosis of AApoA-I and AFib respectively in the Chinese patients, which were inconclusive by IHC alone, and got an accurate typing by multidisciplinary analysis of the detailed clinical evaluation, rational interpretation of IHC, MS-based proteomics, and genetic testing. Our study emphasized the key role of mass spectrometry and genetic analysis in the typing and diagnosis of hereditary amyloidosis.

## Supplementary information

**Additional file 1.** Clinical characteristics

**Additional file 2.** Mass spectrometry data by spectra. The representative mass spectrometry data showed the dominant ApoA-I peptides in case 1 (a) and the fibrinogen Aα chain peptide in case 2 (b) respectively

## Data Availability

All data generated or analyzed in this study are included in the main manuscript and additional supporting files.

## Abbreviations

AA: Amyloid A amyloidosis
ApoA-I: Apolipoprotein A-I
EM: Electron microscopy
ESRD: End-stage renal disease
AFib: Fibrinogen Aα chain amyloidosis
HDL: High-density lipoprotein
AL: Immunoglobulin light chain amyloidosis
IF: Immunofluorescence
IHC: Immunohistochemistry
LC-MS/MS: Liquid chromatography and electrospray tandem mass spectrometry
Lect2: Leukocyte chemotactic factor 2
LMD/MS: Laser microdissection and mass spectrometry
MS: Mass spectrometry
PCR: Polymerase chain reaction
