# Supplementary figures and images for: Typing of hereditary renal amyloidosis presenting with isolated glomerular amyloid deposition
Source: BMC Nephrol. 2019 Dec 23;20:476. doi: 10.1186/s12882-019-1667-5 (PMC6929319; doi:10.1186/s12882-019-1667-5)

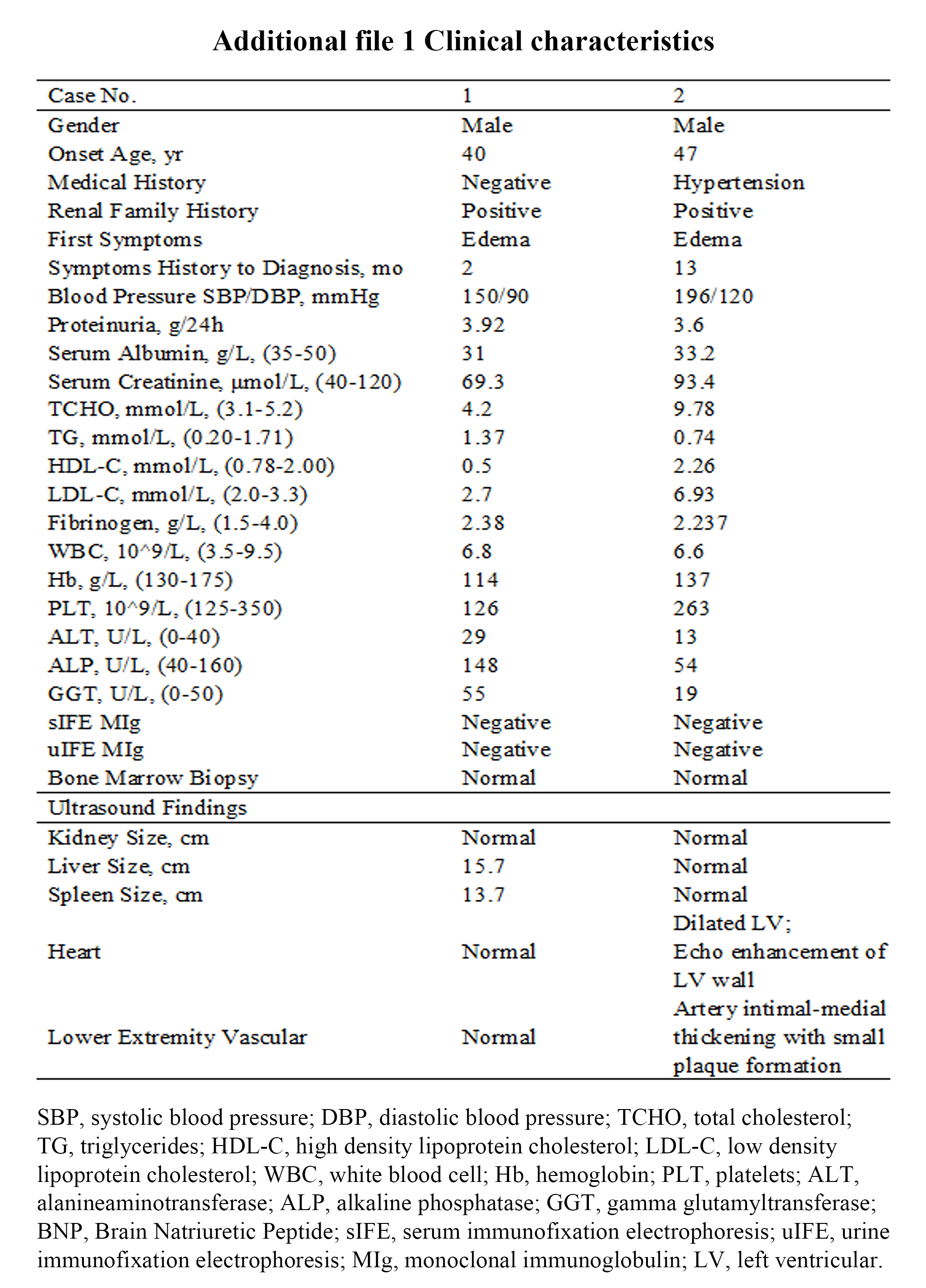

Supplement: Supplementary file 1 — Additional file 1. Clinical characteristics [file 12882_2019_1667_MOESM1_ESM.jpg]

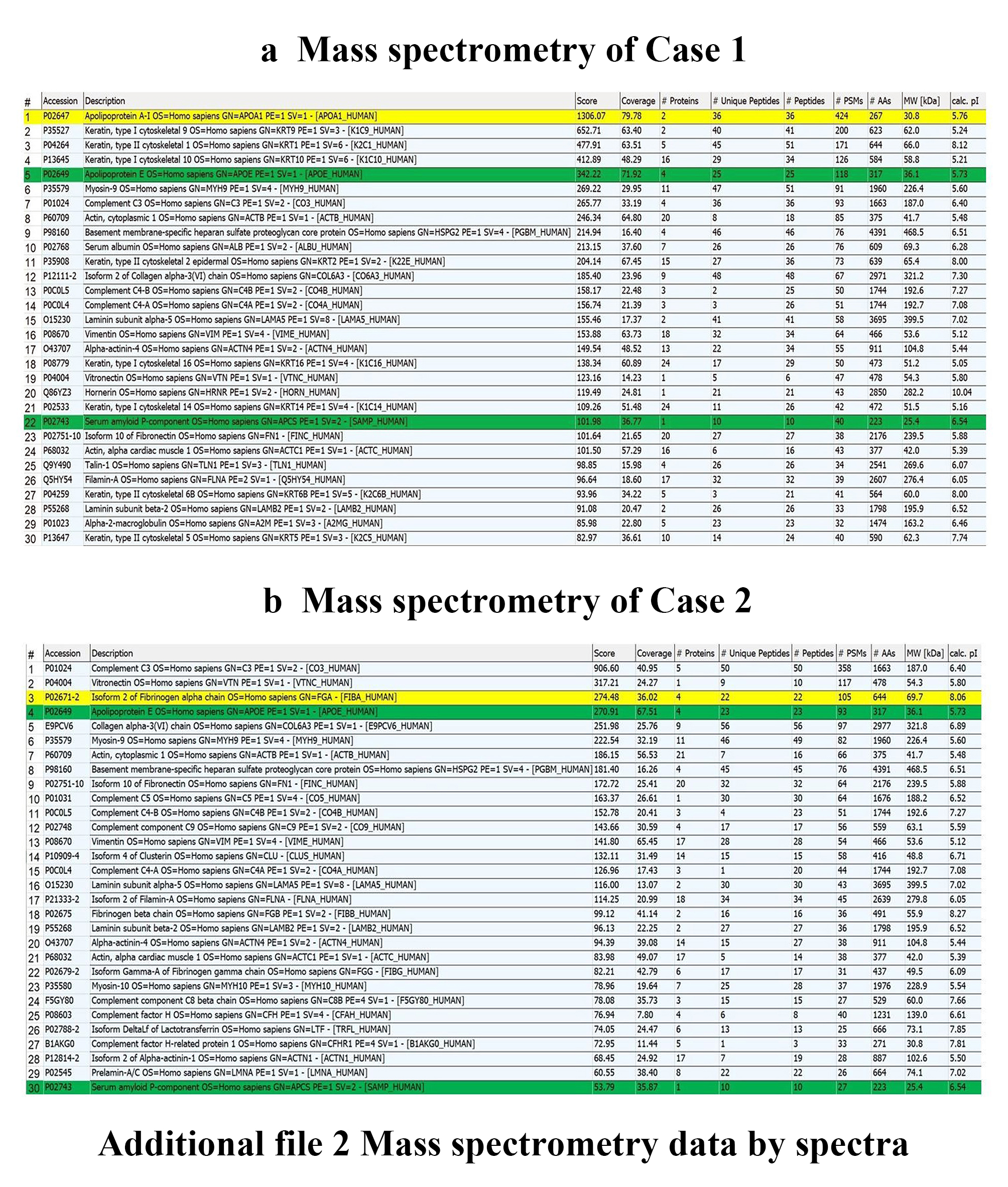

Supplement: Supplementary file 2 — Additional file 2. Mass spectrometry data by spectra. The representative mass spectrometry data showed the dominant ApoA-I peptides in case 1 (a) and the fibrinogen Aα chain peptide in case 2 (b) respectively [file 12882_2019_1667_MOESM2_ESM.jpg]
